# An Effect of the Preliminary Formation of the Balance Concept: The Change in Ways of Elementary Physics Problem Solving

**DOI:** 10.11621/pir.2025.0402

**Published:** 2025-12-01

**Authors:** Elena V. Vysotskaya, Anastasia D. Lobanova, Mariya A. Yanishevskaya

**Affiliations:** a Federal Scientific Center for Psychological and Interdisciplinary Research, Moscow, Russia

**Keywords:** multiplicative concepts, balance task, school subjects propaedeutics, science learning, transition to secondary education

## Abstract

**Background.:**

The classical “lever balance problem” has always been a challenge for students, as it requires special coordination of weight and distance changes. At the core of this coordination lies the “third” magnitude of the multiplicative structure, the torque, which is often overlooked in psychological research on the development of the equilibrium concept.

**Objective.:**

An introductory computer-supported module was developed based on the principles of developmental instruction (Davydov). Our research goal was to demonstrate one of the delayed effects of the preliminary multiplicative concept formation in the fifth grade on students’ acquiring the corresponding topic in elementary physics in the seventh grade.

**Design.:**

The experimental group included 43 seventh-graders who had been taught using our introductory equilibrium curriculum two years prior (4 lessons). The control group (44 seventh-graders) from the same schools had not participated in any special curriculum. The assessment procedure simulated the “physics lesson” with reading the textbook and watching the educational video. Then students had to complete five balance tasks of two types (with one or multiple locations for weights on each lever arm).

**Results.:**

The results revealed that students in the experimental group did significantly better solving the “scattered weights” problems. When the digital simulation allowed all students to resort to trials, the control group relied on the “trial and error” method signiicantly more than the experimental group.

**Conclusion.:**

Students’ adherence to the “conceptual” approach was most clearly revealed through their way of solving “scattered weights” tasks and the number of “excessive” trials in computer simulation. The results proved the lasting effect of our introductory curriculum.

## Introduction

Complex concepts with multiplicative structure are one of the stumbling blocks in the middle school curriculum. The group of scientific concepts related to equilibrium which also have a multiplicative structure are well-known for the difficulties they present for students at the beginning of their school physics studies; thus, there are many studies concerning these diiculties and the ways to acquire the concepts.

The introduction of digital simulations of dynamic objects which students use for their own experimentation has not resolved the issues of forming concept-mediated actions (Tahai et al., 2019; Van der Graaf, 2020). On the contrary, it has made the lack of comprehension of these mechanisms even more evident. As digital simulations of hands-on experiments are introduced for self-guided student inquiry (with less attention paid to the theoretical grounds of the matter presented in textbooks), there is a tendency for students to immerse into chaotic trials, which are meaningless and even inconsistent with previous results — in the same way that students would deal with the “real-life” laboratory equipment. To our mind, such a tendency might explain the students’ poor results taught through “unguided manipulation” conditions in the study by P.N. Chletsos and R. de Lisi (1991), as compared to those students who were taught under “direct mentoring” conditions. The challenge for educational designers is to embed the means to develop the conceptual way of thinking (as presented in textbooks) into work with digital environments — so that the meaningless trials and “roundabout” ways of solving problems would be principally excluded. Within the developmental instruction framework, the concept is acquired by students as a reflection of their own purposeful actions performed according to the concept “under construction”. Th erefore, the analysis of the prerequisites for concept-mediated action formation remains as relevant as ever.

The “classical” research into the way in which students gradually reduce the number of typical mistakes in balance problem solving has revealed a number of intermediate steps in the sequence of their self-guided progression towards semi-effective strategies of evaluation and achievement of equilibrium based on hands-on experimentation. Th ese trajectories, and the pitfalls on the way, as well as the empirically obtained rules are thoroughly described by B. Inhelder and J. Piaget (1958) and subsequent studies (Boom, & ter Laak, 2007; Boom et al., 2001; Li et al., 2017; Normandeau et al., 1989; Siegler, 2013; Siegler, & Chen, 2002; Smith et al., 2023; Xu et al., 2021, etc.).

The essential difficulty of the balance problem stems from the necessity to consider and coordinate two parameters (weight and distance), changing separately, bound by the concept of equilibrium. Children of preschool age often notice the number of weights as seemingly the most reliable parameter. The research on “age-dependent” ability to determine distances as contributing to weight changes on the chosen side has let the issue of inding the right way to deliver the concept a challenge (Boom, & ter Laak, 2007; Jansen, & van der Maas, 2002, Lehtinen et al., 2022). Conflict situations, where students have to compare the weight of heavy objects closer to the fulcrum with those that are lighter but are farther away, cause them to muddle through and fail (Filion, & Sirois, 2021). More complicated tasks where weight units are attached in several positions along the balance scale (scattered weight task) are known to be unsolvable even for adults (Siegler, 2013).

The exact “quantitative” way to consider weight and distance (by multiplication) is counterintuitive. Numerous attempts by students to obtain balance involve sorting through various combinations of tricks to deal with both parameters, such as little-by-little transpositions in configuration of weight units, symmetrical positions, adding distance to weight, counting differences between weights and distances, etc. However, none of these are then transformed into the “4th rule” in Siegler’s terms, and the barrier between empirical guesswork and real understanding seems impenetrable (Lehtinen et al., 2024, p. 151).

The basis for our studies has long been the developmental instruction framework (Davydov, 2008), which focuses on the logical-genetic analysis to trace the origin of the concept that is being formed. According to this approach students are to reconstruct the “matter-related” nature of the concept through their own actions, by means of special modeling, which allows them to use the “raw” concept as a tool of orientation for each operation (Engeness, 2021; Galperin, 1989; Talyzina, 1968). As we implement V.V. Davydov’s and P.Ya. Galperin’s approach to the multiplicative concepts formation, our research has yielded certain promising results (Vysotskaya et al., 2022, 2024a, 2024b).

Like other concepts involving complex structure, at the core of the equilibrium concept appears a certain “third” value that is responsible for the balance of the lever. It is not as “eye-catching” as the previous two, weight and distance, and cannot be made equal directly. The reference to this “third” value, the torque, derived from weight and distance, is common in introductory physics curricula, while the psychology research rarely considers it in the context of the development of the child’s comprehension of the relation between the two parameters when the lever is not balanced (Kalmykova, 1981; Martin, & Rubtsov, 1991,Konokotin, 2023, Lehtinen, 2024, etc.). Th ough the torque is sometimes mentioned (*e.g.,* Filion, & Sirois, 2021; Hardiman et al., 1986; Skyttä et al., 2025) as part of the adult view on the matter, being a “construct that allows prediction of what will happen for any combination of the two variables” (Hardiman et al., 1986, p. 63), it is discarded as specific to teaching physics and not considered as a conceptual tool for students to acquire prior to their formal school studies.

As we investigate the psychological conditions necessary for the development of students’ capacity to think conceptually when solving balance tasks, we assume “equilibrium” (equivalence of total loads pushing the lever in opposite directions) to be the reference point in the concept development that sheds light upon all particular operations with objects on the lever. The load evaluation procedure would thus be responsible for settling the concept origin, and speciically the purposeful preservation and reparation of the balanced state of a lever can be considered as the basis for assimilation of the conceptual “background” of all balance tasks. The learning unit has been designed to support students’ adoption of the load evaluation procedure as the actual means of operating the balance through the “law of the lever”, maintaining the predicted “load value” (the latent third quantity) equal on both sides. This “shift” to the theoretical approach to practical problems allows students to avoid the track of meaningless trials right from the start, but requires providing special support for students’ progress. Within the present research, we focus on the psychological conditions that develop students’ capacity to act conceptually as opposed to the “trial-and-error” method.

## Methods

We have developed an educational computer-supported environment that would scaffold students’ adoption of the specific load evaluation procedure as the basis for choosing operations in particular situations (Vysotskaya et al., 2023, 2024a, 2024b). Our current research goal was to ascertain that the propaedeutic educational module, which reveals the functionality of the load equivalence evaluation, may prevent students from typical mistakes and difficulties when dealing with the divergence of the salient and the conceptual, and from “groping” their way to balance the lever during their irst acquaintance with school physics.

The educational module was aimed at placing the load evaluation at the center of students’ consideration. We assume that students’ reliance on the preliminary load evaluation through special symbolic means would lead to the decrease of students’ “addiction” to solutions through empirical trials.

The initial situation for the designed learning path centered on the balance scattered weights task, despite its complexity and “optionality” in the view of traditional science curriculum designers. This task makes any empirical strategies or rules (Siegler, & Chen, 2002), in particular the “proportional” strategy, insufficient. Thus, the general theoretical method of solving practical problems, which we consider to be the load evaluation procedure, should be irst introduced within these tasks that are usually delayed toward the end of equilibrium learning.

**Figure 1.**
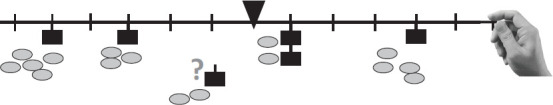
The task: “The lever was balanced, but one weight unit slipped off. Please put it back!”

Special symbolic means were introduced to make explicit the contribution of each weight to be attached to the overall balance according to its mass and distance from the fulcrum. This allowed us to avoid the “empirical” deduction towards the classical “rule of the lever” through practical attempts to balance the lever without appeal to preliminary load evaluation. The actions with tokens representing the units of inflicted load allowed students to plan, choose and control the exact relocation of weights along the balance scale in a series of challenging tasks, without the need for “trial and error” methods, which are often triggered by the availability of lever simulations. It is the reference to the load evaluation that can support solving problems of any level of difficulty (*Figure 1*).

The load evaluation with tokens allows students to solve this and other “counterintuitive” tasks.

Students worked in pairs on the opposite arms of the balance scale, responsible for the weight units’ relocation on their own side. The goal was to achieve equilibrium, which could only be accomplished as a joint result. Therefore, the special modeling of the load using tokens was essential for coordinating students’ further operations through mutual discussion. The participants in joint problem-solving had to carry out the seemingly “excessive” yet indispensable load evaluation procedure and settle the exact load value and the required adjustments of the weight configuration accordingly. Thus, each of them could further adapt his own work with the weight units (adding, removing or shifting them) according to the agreed-upon changes in the total load value.

Challenging task conditions boosted substantial communication among partners and kept the reference to load evaluation in demand. For example, some locations for the weights could be cancelled, numbers of available weights could be strictly defined, weights of a new type could be added along with the old ones, and the available trials on a digital lever could be limited.

This propaedeutic module on equilibrium (two study sessions, four lessons in total), which revealed the essential role of load evaluation in solving balance tasks, was conducted with 43 fifth graders from two Moscow schools. Two years after the propaedeutic module these students arrived to 7th grade and began studying physics.

For our current research, we attempted to assess the effect of the preliminary introduction of students to the concept of multiplicative structure through their own purposeful and concept-mediated solving of practical tasks with peculiar dynamic objects. We assumed that this effect would result in a decrease in the number of false trials in practical situations that required acting “conceptually”.

For this purpose, we designed a special assessment procedure to compare the students from our teaching experiment (43 students who entered an “experimental” group) with other students from the same schools who hadn’t undergone any special introduction in the 5th grade (44 students who formed the “control” group). All participants were in the 7th grade (13-15 years of age) and had not yet been taught the “Lever” topic within their physics curriculum. The current school achievement rate in physics did not difer signiicantly between the two samples.

The diagnostic procedure replicated a usual physics lesson, during which students read the textbook, watched videos with common didactic explanations on the topic and solved a series of six tasks, which instructed them to balance the lever and to check their answers with the computer simulation of the balance scale. The textbook fragment (Peryshkin, Ivanov, 2023, pp. 183-188) contained the information on the “law of the lever”, the inverse proportion, which expressed the conditions of balance, the “moment of force” formula as a product of weight and distance. It also noted that “in case there are more than two forces acting on a lever, the lever is balanced if the sum of the clockwise and counterclockwise moments of the forces are equal” (ibid, p.188). The video repeated and illustrated the information from the textbook and showed examples of solving the balance problems with only one location for weight units on each arm and with scattered weights. The simulation enabled several trials for some of the tasks — thus it did not exclude the possibility of receiving these answers from mere trial-and-error.

**Figure 2.**
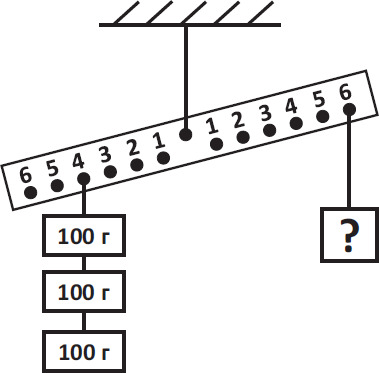
Find the weight of the load that needs to be placed on the sixth position on the right arm to bring the lever into balance

**Figure 3.**
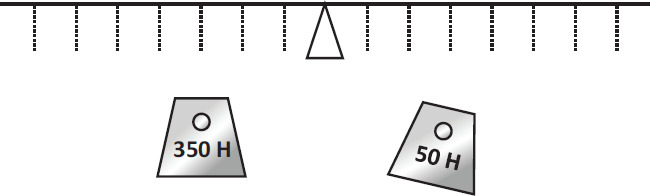
Two weights, 350 H and 50 H, must be balanced on a lever. The larger weight should be placed 4 cm away from the fulcrum. How far from the fulcrum should the opposite arm weight be?

Each task was presented both on paper and on the computer screen in front of each student. Students were supposed to solve each problem and to check the answer on the corresponding digital simulation screen, rearranging the coniguration of weights according to their solution and pushing the “Try!” button to see whether the lever would tip to one side or the other or stay balanced. The number of available trials for each task was displayed on the screen. The first task introduced students to the work with the digital simulation; it was solved by all students and those results were excluded from overall analysis. The next two tasks (*Figures 2 and 3)* required the student to balance the lever with only one location for weights on each arm (tasks with “concentrated weights”).

These two tasks allowed only one attempt to check the solution on the digital lever.

**Figure 4.**
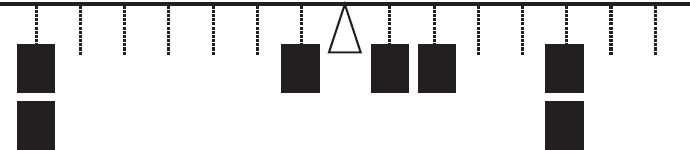
An example of the scattered weights task: How should one of these weights be moved to reach the balance?

The subsequent three tasks presented three different arrangements of scattered weights similar to the one in *Figure 4* (resembling the examples from the instructional video). The first scattered weights task asked whether the lever in the picture was balanced. A single trial was allowed to check the answer. The other two tasks required balancing the given configuration of weights by adding or shifting exactly one weight unit. These two tasks allowed up to six trials.

The task solution was considered correct if the adequate calculations and reasoning in any form had been performed. Students’ work on tasks of both types and the logs of their trials in the computer simulation were analyzed and compared within the two samples.

## Results

**Figure 5.**
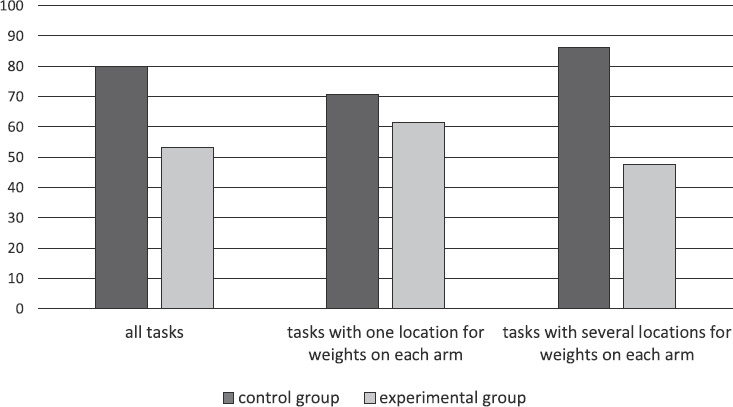
The percentage of the correctly solved tasks in two groups

*Note. The difference in the achievement rate between the experimental and control group is significant (φ = 2.681, p < .01 for all tasks and φ = 3.964, p < .01 for “scattered weights” tasks and φ = .825, p >.05 for “concentrated weights” tasks).*

The data for our analysis consisted of a total of 435 paper-and-pencil solutions, along with corresponding computer log fi les. Th ere were 312 correct solutions (71.7%) where some reasoning was provided. The success rates in the experimental and control group are presented in **Figure 5*.*

The information about the trials completed by students in the process of problem-solving was obtained from the computer logs. According to task conditions, one trial had to be performed in order to check the solution and move to the next task’s screen. We focused on the number of trials performed for the last two tasks, where students were allowed to make more than one checking trial. In this way, a total of 309 trials were recorded by the computer (for the last two tasks), including 174 necessary trials and 135 “excessive” trials. Among them, 23 “excessive” trials were made by students in the experimental group and 112 by students in the control group (χ^2^ = 34.927, p < .01).

**Figure 6.**
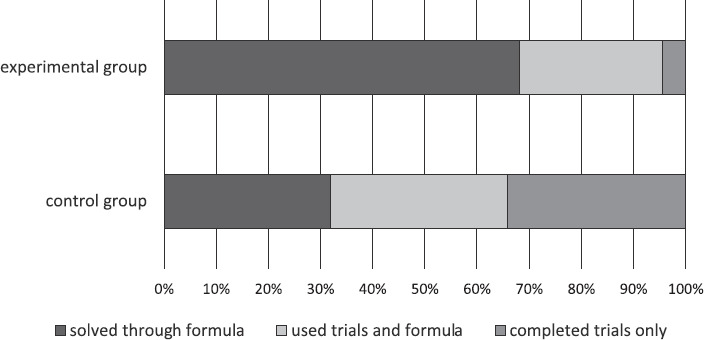
The percentage of students in the experimental and control group who used three different strategies

In order to outline our sample groups, we assigned each student to one of the three clusters, according to the way they solved two of the scattered weights tasks (*Figure 6*):

- those who successfully solved both tasks without any additional trials and provided the relevant reasoning, 44 students (30 of them were among the experimental group, 14 in the control group, φ = 3.637, p < .01). There were no extra trials performed by these students.
- those who made some trials but submitted correct reasoning according to the formula from the textbook in one or both tasks, 26 students (11 of them were members of the experimental group, whereas 15 were from the control group, φ = .867, no significant differences). There were 58 excessive trials done by these students.
- those who tried to obtain the answer through trials only, unable to provide any reasoning according to the textbook or video, no matter whether they succeeded in balancing the lever or not, 17 students (2 of them were in the experimental group and 15 in the control group, φ = 3.777, p < .01). There were 77 extra trials performed by these students.

## Discussion

The results of our assessment showed that students in the experimental group provided significantly more well-reasoned correct solutions than the students in the control group (*Figure 5*, all tasks, scattered weights tasks), which generally indicates the effect of the two propaedeutic study sessions implemented two years prior, according to the instruction described above (Vysotskaya et al., 2023). The students’ performance in tasks of diferent types should now be viewed in detail.

The number of correctly solved “concentrated weights” tasks appeared to be slightly higher in the experimental group than in the control one, but the diference is not as great as in the “scattered weights” tasks (*Figure 5*). We believe that students’ performance in the tasks with “concentrated weights” mainly depended upon students’ ability to absorb the new learning material from our simulated “physics lesson”, which contained sufficient instruction and reasoning for relevant problem solving, rather than on the assimilation of the general way of handling multiplicative magnitudes. Apparently, these tasks (*Figures 2*, *3*) reveal students who managed to grasp the instruction from the textbook and video. We assume that for the same reason the diference between students’ performance in “concentrated” and “scattered weights” tasks in the control group is insigniicant. Moreover, the “concentrated” weights tasks can be solved using simpliied counting strategies with “inverse proportion” or derived formulas described in the textbook, which overlap with “common-sense” springing from everyday experience.

The difference in students’ completion of “scattered weights” tasks (*Figure 4*) is more relevant. Thus, we have additionally analyzed the patterns of students’ solutions in these cases. Apparently, the availability of six trials in the simulation for the last two tasks created new obstacles: some students decided to use these “additional” trials for solving the task, skipping the template calculation of balance from the textbook and instructional video. The computer logs revealed “excessive” trials (23 were performed in the experimental group and 112 were performed in the control group); several trials were testing some incorrect weight conigurations, attempting to reach the balanced state (for example moving one weight gradually) until the trials were exhausted. Sometimes even six trials were not enough to ind the balanced conigu-ration; we even observed the sequences of trials, where each additional step only increased the overweight (12 unsuccessful solutions in total, performed exclusively in the control group). We matched the excessive trials with lack of any reasoning in students’ works and assumed that these students (2 in the experimental group and 15 in the control group, *Figure 6*) resorted to the “trial-and-error” method when they did not see any sense behind the “moment of force” calculations from the lesson materials as they were not apparently connected to the salient features of the situation in front of them.

It is worth noting that should these tasks be solved “theoretically”, i.e. starting with the load evaluation or formula, it would not require any trials at all. The results show that most of the students taught within the propaedeutic curriculum in the fifth grade did not refer to the “trial-and-error” route in order to assess the given weights configuration and found their way to establish the balance (83% correct solutions of three “scattered weights” tasks with one checking trial in the experimental group versus 50% in the control group). On the contrary, a significant number of students from the control group (15 students) did not consider these two tasks from the “theoretical” perspective implied in the textbook, and relied on mere guessing. Thus, our results indicate that the propaedeutic “conceptual” students’ work with the special symbolic tools for preliminary load evaluation is potentially helpful in preventing students from meaningless trials as they face some practical “hands-on” or “digital” tasks on balancing.

## Conclusion

The multiplicative structure of the concepts, which are common in physics, can sig-niicantly impede students’ comprehension of the teacher’s explanations. To better understand their physics teacher’s explanations, students need to develop special skills for handling multiplicative concepts. This includes the ability to work with magnitudes of different origins and predict the results that will be obtained and their future applications. Otherwise, the difficulties that students face in learning physics prompt teachers to provide simpliied “tricks” and “roundabout” ways which would allow students to avoid adopting new concepts, such as the “torque”.

We believe our results indicate the delayed benei t of the practical propaedeutic module that was conducted in the fifth grade. This module introduced special symbolic tools which revealed the “theoretical” role of the concept-to-be-formed in guiding students’ operations. During the initial stages of equilibrium concept formation, the “elusive” “intangible” third value of the load is crucial for designing future operations with real objects on a lever and predicting the yet-to-be-obtained results.

The “traditional” approach to introducing the concept of the lever “from beyond” — based on calculations of two salient parameters — may, in our opinion, lead to some “formalism” in students’ approach to the problem of equilibrium. We observed students who performed both: the “additional” trials and the calculations according to the formulas presented in the instruction (see *Figure 6)*. These trials suggest that students did not understand the functionality of the multiplication formula and needed to resort to practical trials to achieve a balanced coniguration. Perhaps the correct calculation was done “formally”, as the task required.

The concept of “moment of force” (or the “load” as its pre-concept version) implies that changes in its desired value are deined before any operations on the particular configuration of weights are performed. Once this value has been determined, relocating and adding or removing weights can be planned and executed correctly on the first attempt. The “formalistic” approach here means that students must rely on empirical trials to achieve balance.

By setting the “scattered weights” tasks with available hands-on or digital trials as the integrated express assessment tool for this school topic, we can identify students who are formal in their approach to calculations. Through these tasks, we can also draw their attention to the “theoretical” approach to the reasonable application of the torque concept in problem-solving.

We assume that our propaedeutic activity-based instruction, which focused on the functional components of the future school concept, has proved to be useful and productive. As we observed the “delayed” impact of just two intensive study sessions two years ago, most students from the experimental group were able to reconstruct the meaning of the procedures presented in the textbook and resist the temptation to obtain the answer from trial and error, avoiding the “cumbersome” reasoning. Students maintained the “conceptual” approach, which we believe is an important educational gain that will support their future learning in science. According to contemporary research on youth mentality (Gerlich, 2025; Sumskaya & Solomeina, 2022; Vu, 2025; etc.), the development of culturally-mediated, “non-artificial” conceptual thinking is gaining relevance. This is seen as a response to the abundance of pre-digested knowledge characteristic of ubiquitous digitalization.

## Limitations

More sensitive diagnostic tools are needed to monitor the delayed efects of propaedeutic action-based instruction on the development of concepts with a multiplicative structure. Tasks that cannot be solved through a series of “trials” and the meaningless application of formulas, are required to reveal the functionality of these concepts in problem-solving. The design of the propaedeutic modules to support the acquisition of concepts with complex structure also remains a priority. One of the challenges in this regard is the design of specialized support for students’ transition from the propaedeutic introduction to the corresponding school curriculum.

## References

[ref1] Boom, J., Hoijtink, H., & Kunnen, S. (2001). Rules in the balance: Classes, strategies, or rules for the balance scale task? Cognitive Development, 16(2), 717–735. 10.1016/S0885-2014(01)00056-9

[ref2] Boom, J., & ter Laak, J. (2007). Classes in the balance: Latent class analysis and the balance scale task. Developmental Review, 27(1), 127–149. 10.1016/j.dr.2006.06.001

[ref3] Chletsos, P.N., & De Lisi, R. (1991). A microgenetic study of proportional reasoning using balance scale problems. Journal of Applied Developmental Psychology, 12(3), 307–330. 10.1016/0193-3973(91)90003-M

[ref4] Davydov, V.V. (2008). Problems of developmental instruction. Nova Science Publishers. Engeness, I. (2021). P.Y. Galperin’s development of human mental activity: Lectures in educational psychology. Springer Nature. 10.1007/978-3-030-64022-4

[ref5] Filion, V.M., & Sirois, S. (2021). Children’s (mis)understanding of the balance beam (Online Edition). Frontiers in Psychology, 12, 702–524. 10.3389/fpsyg.2021.702524 PMC8419348 34497561

[ref6] Galperin, P.Y. (1989). Organization of mental activity and effectiveness of learning. Soviet Psychology, 27(3), 65–82. 10.2753/RPO1061-0405270365

[ref7] Gerlich, M. (2025). AI tools in society: Impacts on cognitive offloading and the future of critical thinking. Societies, 15(1), 6. 10.3390/soc15010006

[ref8] Hardiman, P.T., Pollatsek, A., & Well, A.D. (1986). Learning to understand the balance beam. Cognition and Instruction, 3(1), 63–86. 10.1207/s1532690xci0301_3

[ref9] Inhelder, B., & Piaget, J. (1958). The growth of logical thinking from childhood to adolescence. Basic Books. 10.1037/10034-000

[ref10] Jansen, B.R., & van der Maas, H.L. (2002). The development of children’s rule use on the balance scale task. Journal of Experimental Child Psychology, 81(4), 383–416. 10.1006/jecp.2002.2664 11890728

[ref11] Kalmykova, Z.I. (1981). Produktivnoe myshlenie kak osnova obuchaemosti [Productive thinking as the basis of learning]. Pedagogika.

[ref12] Konokotin, A.K. (2023). Kommunikatsiia i refleksiia kak usloviia organizatsii detsko-vzroslykh obshch-nostei [Communication and reflection as conditions for organizing child-adult communities]. In V.V. Rubtsov (Ed.), Razvitie kommunikativno-refleksivnykh sposobnostei u detei 6-10 let v zavisi-mosti ot sposobov organizatsii uchebnykh vzaimodeistvii: Kollektivnaia monografía [Development of communicative and reflective abilities in children aged 6-10 years, depending on ways of organizing educational interaction: A collective monograph] (pp. 125–154). MSUPE Publishing House 10.17759/chp.2021170205

[ref13] Lehtinen, A., Nieminen, P., Pehkonen, S., & HähkiÖniemi, M. (2022). Comparing guidance via implicit and explicit model progressions in a collaborative inquiry-based learning environment with different-aged learners. Education Sciences, 12(6), 393. 10.3390/educsci12060393

[ref14] Lehtinen, A., Pehkonen, S., Nieminen, P., & HähkiÖniemi, M. (2024). Collaborative balance rule learning: Do students’ age, group composition, prior knowledge, and scientific reasoning skills matter? Nordina, 20(2). 10.5617/nordina.10186

[ref15] Li, F., Xie, L., Yang, X., & Cao, B. (2017). The effect of feedback and operational experience on children’s rule learning. Frontiers in Psychology, 8, 534. 10.3389/fpsyg.2017.00534 28450838 PMC5390043

[ref16] Martin, L., & Rubtsov, V.V. (1991). The dynamics of collective interaction and its role in the development of understanding in children. Soviet Psychology, 29(3), 66–81. 10.2753/RPO1061-0405290366

[ref17] Normandeau, S., Larivée, S., Roulin, J.L., & Longeot, F. (1989). The balance-scale dilemma: Either the subject or the experimenter muddles through. The Journal of Genetic Psychology, 150(3), 237–250. 10.1080/00221325.1989.9914594 2809572

[ref18] Peryshkin, I.M., & Ivanov, A.I. (2023). Fizika. 7 klass. Bazovyi uroven’ [Physics. 7th grade. Basic level] Prosveshchenie.

[ref19] Siegler, R. (2013). Children’s thinking: What develops? Lawrence Erlbaum Associates.

[ref20] Siegler, R.S., & Chen, Z. (2002). Development of rules and strategies: Balancing the old and the new. Journal of Experimental Child Psychology, 81(4), 446–457. 10.1006/jecp.2002.2666 11890730

[ref21] Skyttä, S., Lehtinen, A., Nieminen, P., & HähkiÖniemi, M. (2025). Exploring the connection between sixth grade students’ adaptive number knowledge and proportional rules in balance scale problems. International Journal of Science and Mathematics Education, 1–19. 10.1007/s10763-025-10563-w

[ref22] Smith, K., Battaglia, P., & Tenenbaum, J. (2023). Integrating heuristic and simulation-based reasoning in intuitive physics. PsyArXiv. 10.31234/osf.io/bckes

[ref23] Sumskaya, A., & Solomeina, V. (2022). Russian Media Generation of the “Digital Borderline” Theoretical Reflection and Empirical Verification. World of Media. Journal of Russian Media and Journalism Studies, 4, 68–93. 10.30547/worldofmedia.4.2022.4

[ref24] Tahai, L., Wallace, J.R., Eckhardt, C., & Pietroszek, K. (2019). Scalebridge: Design and evaluation of adaptive difficulty proportional reasoning game for children. In 2019 11th International Conference on Virtual Worlds and Games for Serious Applications (VS-Games) (pp. 1–4). IEEE. 10.1109/VS-Games.2019.8864526

[ref25] Talyzina, N.F. (1968). The stage theory of formation of mental operations and the problem of the development of thought. Soviet Education, 10(4), 38–42. 10.2753/RES1060-9393100438

[ref26] Van der Graaf, J. (2020). Inquiry-based learning and conceptual change in balance beam understanding. Frontiers in Psychology, 11, 1621. 10.3389/fpsyg.2020.01621 32774317 PMC7387727

[ref27] Vu, T.V. (2025). Vietnamese media and communication students’ news consumption in the digital age. World of Media, Journal of Russian Media and Journalism Studies, 2, 73–95. 10.30547/worldofmedia.2.2025.4

[ref28] Vysotskaya, E.V., Lobanova, A.D., & Yanishevskaya, M.A. (2022). Mastering models in a quasi-learning situation of problem-solving. Psychological Science and Education, 27(1), 27–36. 10.17759/pse.2022270103

[ref29] Vysotskaya, E.V., Lobanova, A.D., & Yanishevskaya, M.A. (2023). Propedevticheskii uchebnyi modul’ “Ravnovesie”: podderzhka kollektivno-raspredelennoi uchebnoi deiatel’nosti v formirovanii kog-nitivnykh i kommunikativnykh metapredmetnykh rezul’tatov [Propaedeutic educational module “Equilibrium”: support for joint learning activities in the formation of cognitive and communicative meta-subject results]. Author’s Club.

[ref30] Vysotskaya, E., Yanishevskaya, M., & Lobanova, A. (2024a). The features of modeling mediation in digital support for formation of multiplicative concepts. Psychology in Russia: State of the Art, 17(2), 100–113. 10.11621/pir.2024.0207 PMC11562006 39552780

[ref31] Vysotskaya, E., Yanishevskaya, M., Lobanova, A., & Taysin, M. (2024b). Scafolding multiplicative concepts formation: A way of digital support. In A. Veraksa & Yu. Solovieva (Eds.), Learning mathematics by cultural-historical theory implementation: Understanding Vygotsky’s approach (pp. 165–182). Springer Nature. 10.1007/978-3-031-66894-4_10

[ref32] Xu, L., Ferguson, J., & Tytler, R. (2021). Student reasoning about the lever principle through multimodal representations: A socio-semiotic approach. International Journal of Science and Mathematics Education, 19, 1167–1186. 10.1007/s10763-020-10102-9

